# RNA Demethylase FTO Uses Conserved Aromatic Residues to Recognize the mRNA 5′ Cap and Promote Efficient m^6^A_m_ Demethylation

**DOI:** 10.1016/j.jbc.2026.113573

**Published:** 2026-09-17

**Authors:** Brittany Shimanski, Juan F. Marín, Marcin Warminski, Kasun Dilshan Abeyrathne Eluwawalage, Lucas O. Calzini, Riley M. McKeon, Joanna Kowalska, Jacek Jemielity, Jodi A. Hadden-Perilla, Jeffrey S. Mugridge

**Affiliations:** 1 Department of Chemistry & Biochemistry, University of Delaware, Newark, Delaware, USA; 2 Division of Biophysics, Institute of Experimental Physics, Faculty of Physics, University of Warsaw, Warsaw, Poland; 3 Centre of New Technologies, University of Warsaw, Warsaw, Poland

**Keywords:** RNA demethylation, RNA modification, RNA–protein interaction, molecular dynamics, enzyme kinetics, enzyme catalysis

## Abstract

The RNA demethylase FTO acts as a methyl “eraser” to remove either internal *N*^6^-methyladenosine (m^6^A) or 5′ end *N*^6^,2′-O-dimethyladenosine (m^6^A_m_) modifications on mRNA. FTO has an intrinsic preference and significantly faster demethylation rates *in vitro* for m^6^A_m_ modifications located at the 5′ mRNA cap structure, but the structural basis for FTO’s ability to discriminate m^6^A *versus* m^6^A_m_ modifications has remained unknown. Here, we utilize molecular dynamics simulations of FTO–RNA cap complexes to identify conserved aromatic residues on the surface of FTO involved in 5′ cap recognition. Subsequent mutagenesis and enzymology experiments validate the specificity of these residues in engaging the 5′ cap structure to promote m^6^A_m_ demethylation. We also identify a nonpolar surface on FTO that interacts with the 2′-O-methyl group of m^6^A_m_ to impact demethylation kinetics. This work provides the first structure-level insights into how FTO selectively catalyzes m^6^A_m_*versus* m^6^A demethylation on mRNA and advances our understanding of how FTO activity is regulated by diverse mechanisms to help control the epitranscriptome.

*N*^6^-methyladenosine (m^6^A) is the most abundant internal chemical modification of eukaryotic mRNA (1, 2). Installed cotranscriptionally by the METTL3/14 methyltransferase “writer” complex, m^6^A can be found throughout the length of mRNA transcripts and is enriched near stop codons and in the 3′ UTR (3, 4). This widely important modification has impacts on mRNA splicing (5, 6, 7), export (8, 9), translation (10, 11, 12, 13), and stability (14, 15, 16). Notably, m^6^A plays a crucial role in the regulation of mammalian stem cell differentiation (17) and is involved in controlling the expression of human oncogenes and tumor suppressor genes (18, 19, 20). Another highly abundant mRNA methyl modification, *N*^6^,2′-O-dimethyladenosine (m^6^A_m_), is found exclusively at the 5′ end of eukaryotic mRNAs in up to 30% of mammalian 5′ mRNA caps (21). m^6^A_m_ is installed by the writer enzyme CAPAM (or PCIF1), which carries out *N*^6^-methylation of 2′-O-methyladenosine (A_m_) modifications (22, 23, 24, 25). While the functions of m^6^A_m_ modifications remain incompletely defined and may vary by cellular context, recent work suggests these 5′ cap modifications may influence mRNA stability, translation, and transcription, whereas m^6^A_m_ modifications in small nuclear RNA (snRNA) may help regulate snRNA biogenesis and splicing (26, 27, 28, 29, 30, 31, 32, 33). Together, the highly abundant methyl modifications m^6^A and m^6^A_m_ help control mRNA fate and function across the transcriptome.

Fat mass and obesity-associated protein (FTO) is an Fe(II)/2-oxoglutarate-dependent dioxygenase enzyme that acts as an m^6^A demethylase or “eraser”, converting m^6^A or m^6^A_m_ modifications to A or A_m_, respectively, within cellular mRNA transcripts (34, 35, 36, 37). Along with other AlkB homologs in this family (*e.g.* ALKBH5), FTO allows for reversible m^6^A and m^6^A_m_ modification and the dynamic regulation of RNA methylation. Although FTO likely only acts on a subset of m^6^A/m^6^A_m_ modifications found on RNA (33, 38, 39), FTO is linked to splicing (7, 27, 40), cellular metabolism (41, 42, 43, 44, 45, 46), and mammalian development (47, 48, 49). Furthermore, FTO and its methyl eraser functions have close links to the progression of human cancers, such as acute myeloid leukemia (50, 51), colorectal cancer (52, 53), and glioblastoma (54, 55). More broadly, FTO can promote oncogenesis and immune evasion in different cancer contexts (56, 57, 58).

Although FTO has the ability to demethylate both internal m^6^A and 5′ cap m^6^A_m_ RNA modifications, previous studies have shown that FTO has a strong intrinsic preference *in vitro* for m^6^A_m_ demethylation (26) and more recent studies call into question whether FTO truly targets m^6^A modifications in the cell (59, 60). Remarkably, FTO has ∼100-fold greater catalytic efficiency for demethylating m^6^A_m_ than m^6^A modifications on RNA oligonucleotides (26). Furthermore, in model RNA substrates where the 5′ *N*^7^-methylguanosine (m^7^G) and/or triphosphate groups of the eukaryotic cap are removed, FTO-mediated demethylation of m^6^A_m_ is significantly impaired, suggesting that FTO somehow specifically recognizes the 5′ cap structure to promote m^6^A_m_ demethylation. An existing structure of 6mA–ssDNA in complex with FTO provides information about how the m^6^A base is accommodated in the FTO active site and how DNA (and likely RNA) binds a basic cleft on the surface of FTO (37). However, to date there is no structural or biophysical information about how FTO preferentially demethylates 5′ m^6^A_m_ modifications, how FTO might specifically recognize the eukaryotic 5′ cap structure and m^7^G or m^6^A_m_ bases, or the general mechanism(s) by which FTO selectively catalyzes removal of m^6^A_m_ *versus* m^6^A modifications on RNA.

Here, we combine molecular dynamics (MD) simulations of FTO–m^6^A_m_ cap complexes with mutagenesis and enzymology experiments to identify conserved aromatic residues on the surface of FTO involved in 5′ mRNA cap recognition and selective m^6^A_m_ demethylation. We also identify nonpolar residues located just outside of the FTO active site that interact with the m^6^A_m_ 2′-O-methyl group to further mediate m^6^A_m_ demethylation. These experiments provide the first atomic-level information about how 5′ cap recognition promotes m^6^A_m_ demethylation by FTO and give new insights into the mechanisms controlling FTO-mediated mRNA demethylation selectivity.

## Results

### MD simulations identify conserved aromatic FTO surface residues involved in FTO–m^7^G cap interactions

Previous biochemical studies have shown that FTO more efficiently demethylates 5′ m^6^A_m_ modifications as compared to internal m^6^A modifications on RNA (26). Additionally, removing the m^7^G portion of the 5′ mRNA cap structure on model RNA substrates results in a 50% reduction in m^6^A_m_ demethylation activity, and further removal of the cap triphosphate group reduces demethylation by another 40%. These data suggest that there must be FTO residues that selectively recognize the m^7^G and triphosphate groups adjacent to m^6^A_m_ and that these surfaces help to recruit and/or position 5′ m^6^A_m_ modifications for demethylation (26). To elucidate which residues and surfaces of FTO are interacting with the 5′ mRNA cap structure, we performed microsecond MD simulations of FTO in complex with the small 5′ cap molecule m^7^Gppp(m^6^A_m_)pG (Fig. 1*A*) in triplicate. Molecular mechanics/generalized Born surface area (MM/GBSA) calculations were applied to the MD trajectories to identify residues that may contribute to m^7^Gppp(m^6^A_m_)pG binding or recognition (Fig. S1). Of these, three conserved aromatic residues on the surface of FTO were observed to make high-frequency contacts (≤ 3.5 Å) with the 5′ m^7^G base or ribose (Figs. 1*B* and S2): Y220 (Fig. 1*C*), H232 (Fig. 1*D*), and W278 (Fig. 1*E*).

**Figure 1:**
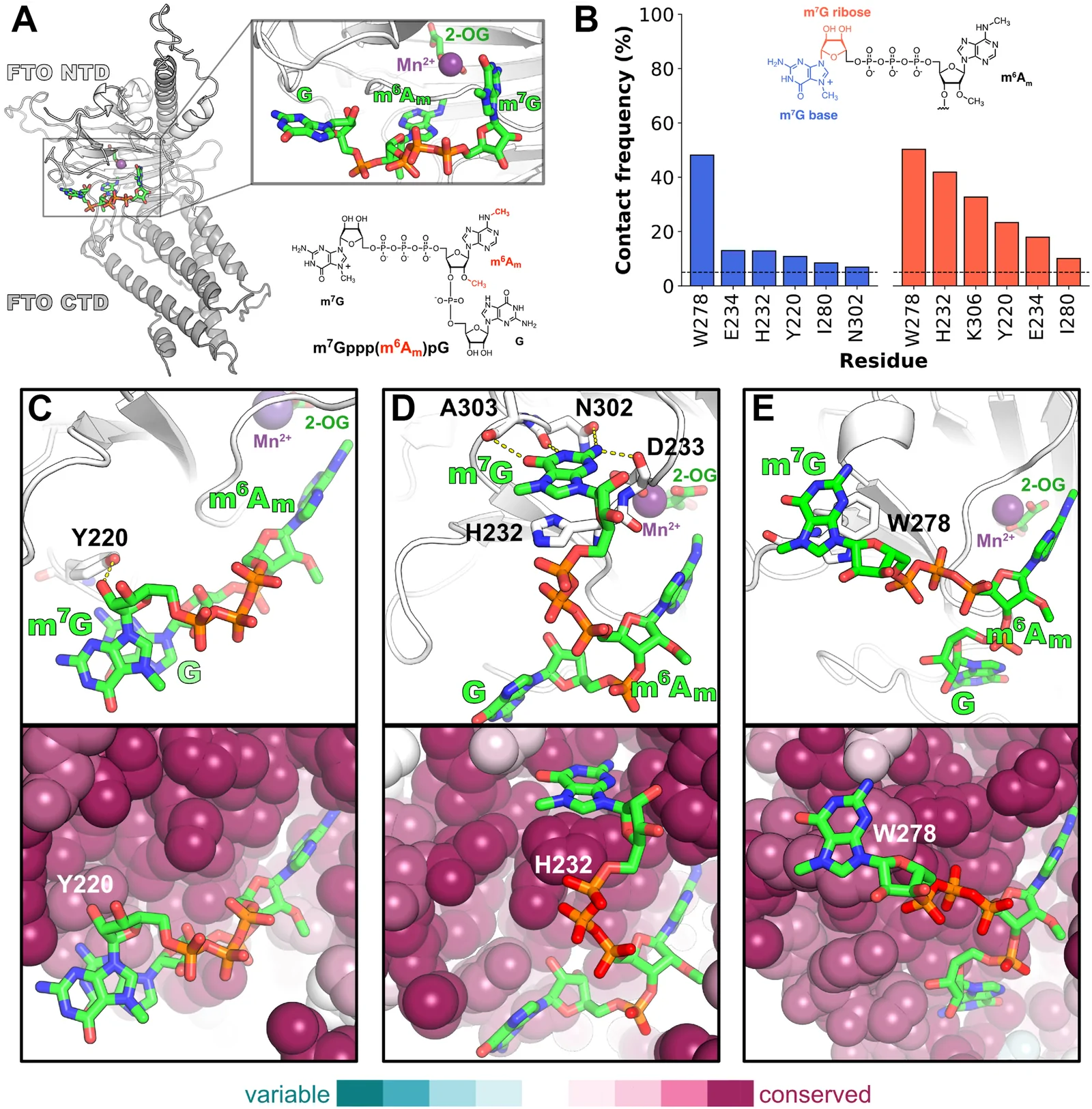
**MD reveals FTO residues with high-frequency FTO–m^7^G interactions.***A,* to provide a starting point for MD simulations, an m^7^Gppp(m^6^A_m_)pG cap structure was positioned on FTO by aligning the m^6^A_m_ base into the FTO active site based on previously determined 6mA-ssDNA-FTO structure PDB 5ZMD. *B,* m^7^G-FTO residue contacts ≤ 3.5 Å with > 5% interaction frequency from MD simulations on the microsecond timescale. Only FTO residues with favorable contributions to the computationally predicted binding affinity (ΔG ≤ −0.25 kcal/mol, Fig. S1) are considered. *C,* representative MD frame showing hydrogen-bonding interaction (*dashed yellow line*) between FTO residue Y220 and m^7^G ribose of the cap structure (*top*). ConSurf (84, 85) analysis shows this is a strongly conserved surface of FTO (*bottom*). *D,* representative MD frame showing m^7^G stacking with conserved FTO residue H232 and hydrogen bonding with N302, A303, and D233 side chain and backbone atoms on FTO surface; hydrogen bonds are shown as *dashed yellow lines* (*top*). In this MD-predicted binding mode, m^7^G interacts with a highly conserved surface of FTO (*bottom*). *E,* representative MD frame showing m^7^G stacking on strongly conserved FTO residue W278. FTO, fat mass and obesity-associated protein; MD, molecular dynamics.

MD simulations show that Y220 can interact with the m^7^G ribose, forming hydrogen bonds through its hydroxyl group (Fig. 1*C*). The aromatic ring of Y220 was not observed to participate in guanine base stacking unless m^6^A_m_ adopted a shallow, catalytically ineffective position in the FTO active site (Fig. S3). The H232 imidazole ring can form a cation-π/stacking interaction with the positively charged m^7^G, while the base makes simultaneous hydrogen bond contacts with backbone atoms of FTO residues N302, A303, and D233, as well as the N302 side chain (Fig. 1*D*). Alternatively, H232 can make CH-π or hydrogen bond contacts with the m^7^G ribose or interact with the triphosphate bridge (ppp). H232 is located on the surface of FTO, immediately adjacent to metal-binding residues in the FTO active site and was treated as neutral during MD simulations. Finally, W278, located on a flexible loop of FTO, can form a cation-π/stacking interaction with the positively charged m^7^G base (Fig. 1*E*).

Each of these three aromatic FTO residues (Y220, H232, W278) are strongly conserved (Fig. 1, *C*–*E*, bottom) and MD simulations show they can engage with the 5′ cap structure in multiple binding modes, including modes that simultaneously involve two of the aromatic residues. For example, H232 can interact with the m^7^G ribose while W278 stacks with the m^7^G guanosine base (Fig. S4*A*), or W278 and Y220 can sandwich m^7^G (Fig. S4*B*).

### Mutations to conserved aromatic surface residues on FTO specifically impair m^6^A_m_ demethylation

To experimentally validate the FTO–m^7^G interactions predicted by MD simulations, we mutated key, high-frequency m^7^G-interacting FTO residues to alanine and compared WT *versus* mutant demethylation activities on both an internal m^6^A-containing RNA substrate and the model cap substrate m^7^Gppp(m^6^A_m_)pG (Fig. 2*A*). Model cap substrates were used in these initial screening experiments to maximize the likelihood that defective m^7^G or cap recognition in mutant FTO constructs results in a strong and observable effect on demethylation activity. LC-MS–based demethylation activity assays were conducted on the panel of FTO mutants to quantify relative demethylation activity after 150 min compared to WT FTO. These biochemical assays showed that FTO Y220A mutation negatively impacted both m^6^A and m^6^A_m_ demethylation; H232A, N302A, W278A, and H232A/W278A mutations had little to no defect on demethylation of 9-mer m^6^A RNA substrate (Fig. 2*A*, “m^6^A-RNA”); while H232A, W278A, and H232A/W278A mutants all significantly impaired demethylation of m^6^A_m_ in the model cap substrate (Fig. 2*A*, “m^6^A_m_ cap”). Time courses with FTO mutants showed similar defects in m^6^A_m_ demethylation kinetics with H232A and W278A mutants (Fig. S5). Surprisingly, N302A had ∼2-fold higher activity on m^6^A_m_ cap than WT FTO. Notably, MD simulations showed that in the absence of m^7^G interactions, N302 and H232 frequently form hydrogen bonds with each other; loss of the N302 side chain may therefore increase accessibility of H232 for cap recognition, consistent with increased activity in the N302A mutant.

**Figure 2:**
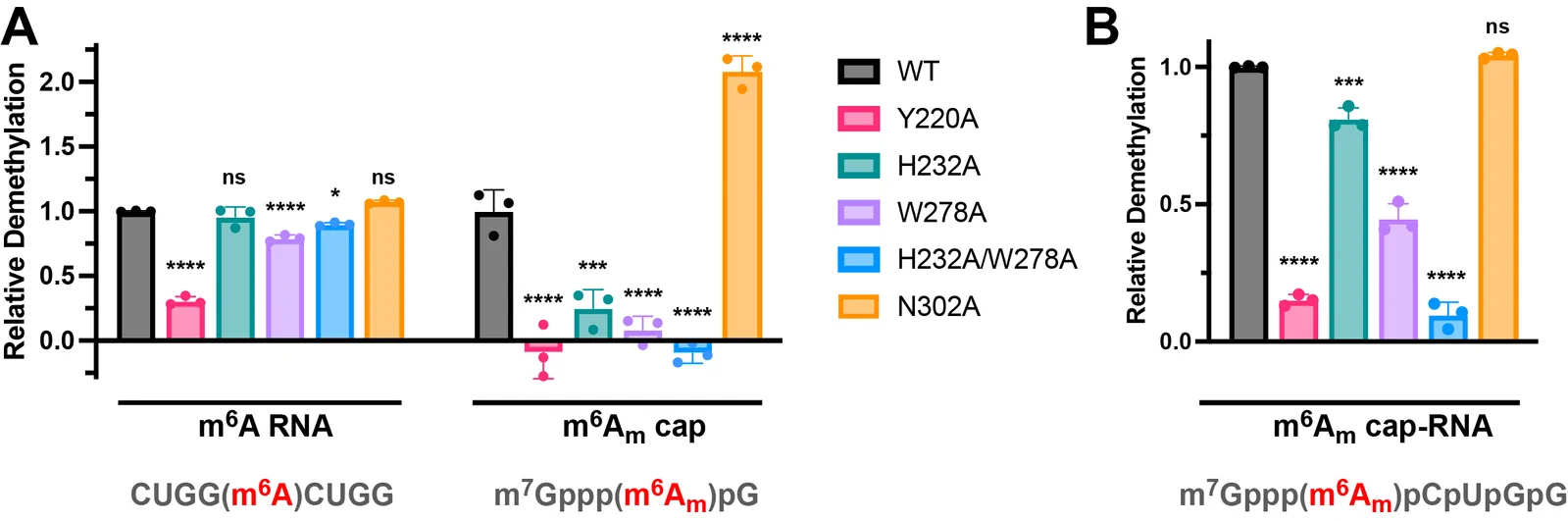
**Enzymatic assays validate FTO residues important for m^6^A_m_ demethylation.***A,* mutation of conserved aromatic residues in FTO identified in MD simulations as m^7^G interaction sites show altered m^6^A_m_ demethylation activity. Endpoint demethylation activity assays were conducted in triplicate with 2 μM CUGG(m^6^A)CUGG (m^6^A-RNA) or m^7^Gppp(m^6^A_m_)pG (m^6^A_m_ cap) and 1 μM FTO enzyme. At 0 and 150 min, reaction samples were quenched with EDTA, digested to single nucleosides with RNAase cocktail, and the relative amounts of m^6^A or m^6^A_m_ were quantified by UHPLC-MS. *B,* mutation of conserved aromatic residues in FTO identified as m^7^G interaction sites show reduced m^6^A_m_ demethylation activity with a longer m^6^A_m_ cap RNA substrate. Endpoint demethylation activity assays were conducted with 2 μM m^7^Gppp(m^6^A_m_)pCpUpGpG (m^6^A_m_ cap-RNA) and 0.1 μM FTO enzyme in triplicate. At 0 and 150 min, reaction samples were quenched with EDTA, decapped, digested to single nucleosides with RNAase cocktail, and the relative amounts of m^6^A_m_ were quantified by UHPLC-MS. For both (*A*) and (*B*), statistical significance was calculated using a one-way ANOVA with Dunnett’s multiple comparisons test (ns *p* > 0.05, ∗*p* ≤ 0.05, ∗∗*p* ≤ 0.01, ∗∗∗*p* ≤ 0.001, and ∗∗∗∗*p* ≤ 0.0001); individual data points are shown for each independent assay replicate (n = 3) and bar graphs are shown as mean values ± SD. FTO, fat mass and obesity-associated protein; m^6^A_m_, *N*^6^,2′-O-dimethyladenosine; MD, molecular dynamics.

We next tested the relative m^6^A_m_ demethylation activities for WT *versus* mutant FTO constructs on the longer capped model RNA substrate m^7^Gppp(m^6^A_m_)pCpUpGpG (“m^6^A_m_ cap-RNA”), which more closely mimics a capped RNA. With this longer m^6^A_m_-containing substrate we observed a significant defect in demethylation activity with Y220A mutation, a small but measurable defect on m^6^A_m_ demethylation for mutation of FTO H232, and, very similar to the results above with the shorter cap substrate, large defects in demethylation activity with W278A and H232A/W278A mutants (Fig. 2*B*). Additionally, Michaelis–Menten kinetic analysis of WT FTO *versus* H232A/W278A FTO (Fig. S6, Table S1) indicated that the impaired m^6^A_m_ demethylation activity of the H232A/W278A mutant primarily reflects a reduction in *k*_cat_, rather than a change in *K*_M_. The mutant exhibited a ∼4-fold decrease in *k*_cat_, whereas *K*_M_ increased by only ∼1.5-fold and was not statistically different from WT (Table S1). Together, these kinetic data show that mutation of FTO residues H232 and W278 significantly impair FTO-mediated demethylation activity of capped m^6^A_m_-containing RNAs, but not internal m^6^A-containing RNAs, suggesting these two conserved FTO surface residues play a key role in recognizing the 5′ cap to promote efficient m^6^A_m_ demethylation catalysis. While Y220A mutation also impairs m^6^A_m_ demethylation, it has similar defects on m^6^A demethylation, suggesting that this mutation more generally impairs FTO activity, folding/stability, and/or RNA binding.

### FTO residues H232 and W278 specifically recognize m^7^G of cap

The MD simulations predicted key conserved residues on the surface of FTO that have high-frequency interactions with the m^7^G group of the RNA 5′ cap, and our biochemical validation experiments showed that mutation of these residues impairs m^6^A_m_ demethylation. We next asked if removal of the m^7^G group from our model cap-RNA substrates eliminates the relative defect in m^6^A_m_ demethylation activity between FTO WT and mutants—as would be expected for FTO residues that specifically and exclusively recognize m^7^G. In demethylation assays with ppp(m^6^A_m_)pCpUpGpG (“ppp-m^6^A_m_-RNA”, Fig. 3*A*), we found that while FTO Y220A still has a strong defect in demethylation activity, H232A, W278A, and H232A/W278A mutants no longer have significant defects in activity compared to WT on this substrate that lacks m^7^G. To further confirm this, we compared time courses for m^6^A_m_ demethylation for WT *versus* H232A/W278A FTO with the normal m^7^G-containing m^6^A_m_-RNA substrate (Fig. 3*B*) and with the m^7^G-lacking ppp-m^6^A_m_-RNA substrate (Fig. 3*C*). Consistent with the endpoint demethylation assays, these experiments show that the FTO H232A/W278A mutant has a dramatic defect in m^6^A_m_ demethylation activity *only when m*^*7*^*G is present* in the substrate; removal of m^7^G lowers the overall demethylation activity, but results in WT and H232A/W278A having similar m^6^A_m_ demethylation rates. Together, these data strongly suggest that FTO H232 and W278 are responsible for selective recognition of the m^7^G group of the 5′ cap structure to promote m^6^A_m_ demethylation, because mutation of these residues results in demethylation defects only on m^7^G-containing m^6^A_m_ cap substrates—but not on internal m^6^A-containing substrates or m^6^A_m_ cap substrates that lack m^7^G.

**Figure 3:**
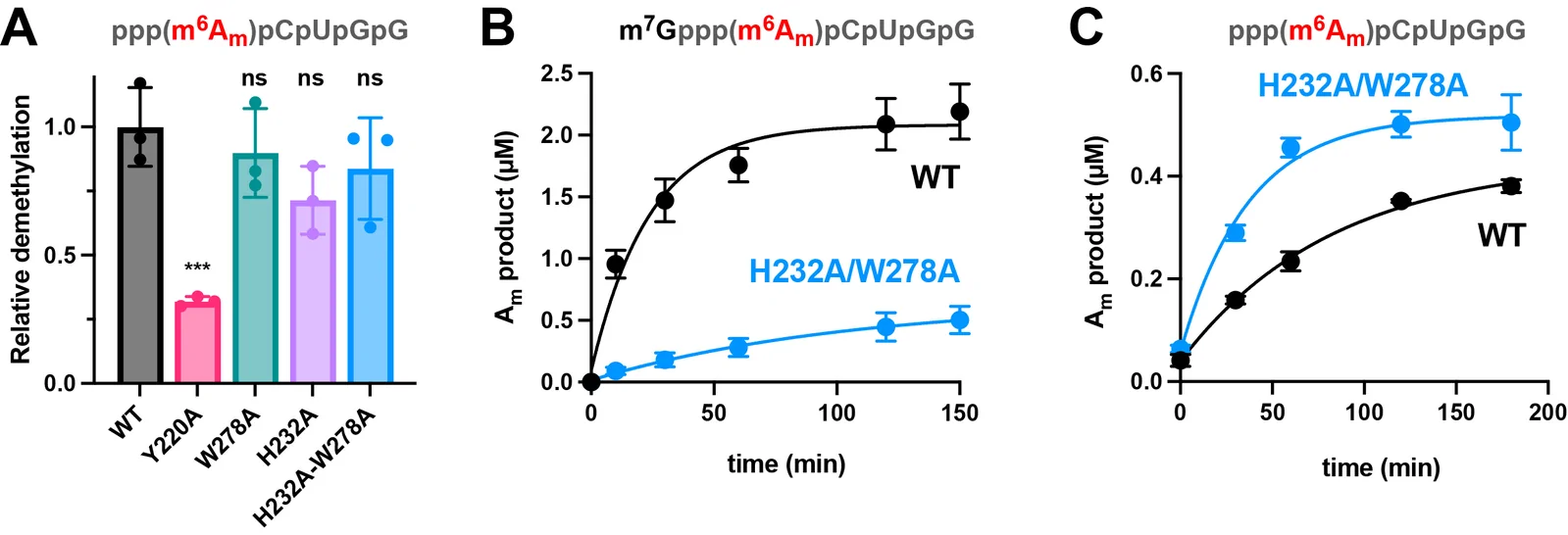
**Demethylation assays with truncated caps implicate FTO residues H232 and W278 in selective m^7^G recognition.***A,* endpoint demethylation assays comparing the relative m^6^A_m_ demethylation activity for WT *versus* mutant FTO constructs with the m^7^G-lacking ppp(m^6^A_m_)pCpUpGpG substrate. Triplicate demethylation assays were carried out as in Figure 2, with 2 μM ppp(m^6^A_m_)pCpUpGpG substrate and 1 μM FTO enzyme. FTO Y220A has impaired m^6^A_m_ demethylation activity on the truncated, m^7^G-less substrate, whereas H232A, W278A, and H232A/W278A mutants have no significant defect and similar activity to WT. Statistical significance was calculated using a one-way ANOVA with Dunnett’s multiple comparisons test (ns *p* > 0.05, ∗∗∗*p* ≤ 0.001); individual data points are shown for each technical replicate (n = 3) and bar graphs are shown as mean values ± SD. *B,* time courses comparing kinetics of A_m_ product formation for WT *versus* H232A/W278A FTO with m^6^A_m_ cap substrate m^7^Gppp(m^6^A_m_)pCpUpGpG show that the FTO H232A/W278A double mutant has significantly impaired demethylation kinetics compared to WT. *C,* time courses comparing kinetics of A_m_ product formation for WT *versus* H232A/W278A FTO with truncated m^6^A_m_ cap substrate ppp(m^6^A_m_)pCpUpGpG, which lacks the m^7^G group, show that the FTO H232A/W278A double mutant has no defect in demethylation kinetics compared to WT. For both (*B*) and (*C*), time courses were carried out in triplicate and data for each time point is shown as the mean value of the independent replicates (n = 3) ± SD. FTO, fat mass and obesity-associated protein; m^6^A_m_, *N*^6^,2′-O-dimethyladenosine; m^7^G, *N*^7^-methylguanosine.

### MD simulations and kinetic assays identify FTO residues important for recognition of the 2′-O-methyl group in m^6^A_m_

Similar to m^7^G, the 2′-O-methyl group on adenosine (A_m_) in the m^6^A_m_ cap structure has also been shown to be important for promoting FTO-mediated demethylation of m^6^A_m_ (26). *In vitro* activity studies indicate that absence of the A_m_ 2′-O-methyl group in model m^7^Gppp(m^6^A)-RNA substrates results in a 50% decrease in FTO’s ability to demethylate the 5′ m^6^A group as compared to m^6^A_m_. However, there is no structural information about how FTO recognizes the A_m_ 2′-O-methyl group to help promote m^6^A_m_ demethylation. To explore this FTO–m^6^A_m_ interaction, we surveyed A_m_ methyl-FTO contacts ≤ 3.5 Å over all frames in the FTO- m^6^A_m_ MD simulations and identified two FTO residues, L109 and I85, which contact the A_m_ 2′-O-methyl group with very high frequencies (Figs. 4*A* and S7). These strongly conserved nonpolar residues are located just outside the active site pocket and provide a hydrophobic surface on which the A_m_ 2′-O-methyl group frequently rests throughout the majority of the MD simulations (Fig. 4, *B* and *C*). Demethylation activity assays comparing WT FTO to L109A or I85A mutants revealed that while both of these mutations resulted in only small defects to m^6^A demethylation on an internal m^6^A-RNA, L109A resulted in a nearly 90% reduction in m^6^A_m_ cap demethylation activity (Fig. 4*D*). Surprisingly, the FTO I85A mutation resulted in a ∼50% increase in m^6^A_m_ cap demethylation relative to WT, suggesting this mutation may open up additional space on this surface or a shallow pocket to accommodate the A_m_ 2′-O-methyl group. Together, these experiments suggest that this nonpolar surface on FTO (L109, I85) plays a key role in interacting with the 2′-O-methyl group of the m^6^A_m_ base to help mediate N^6^ demethylation.

**Figure 4:**
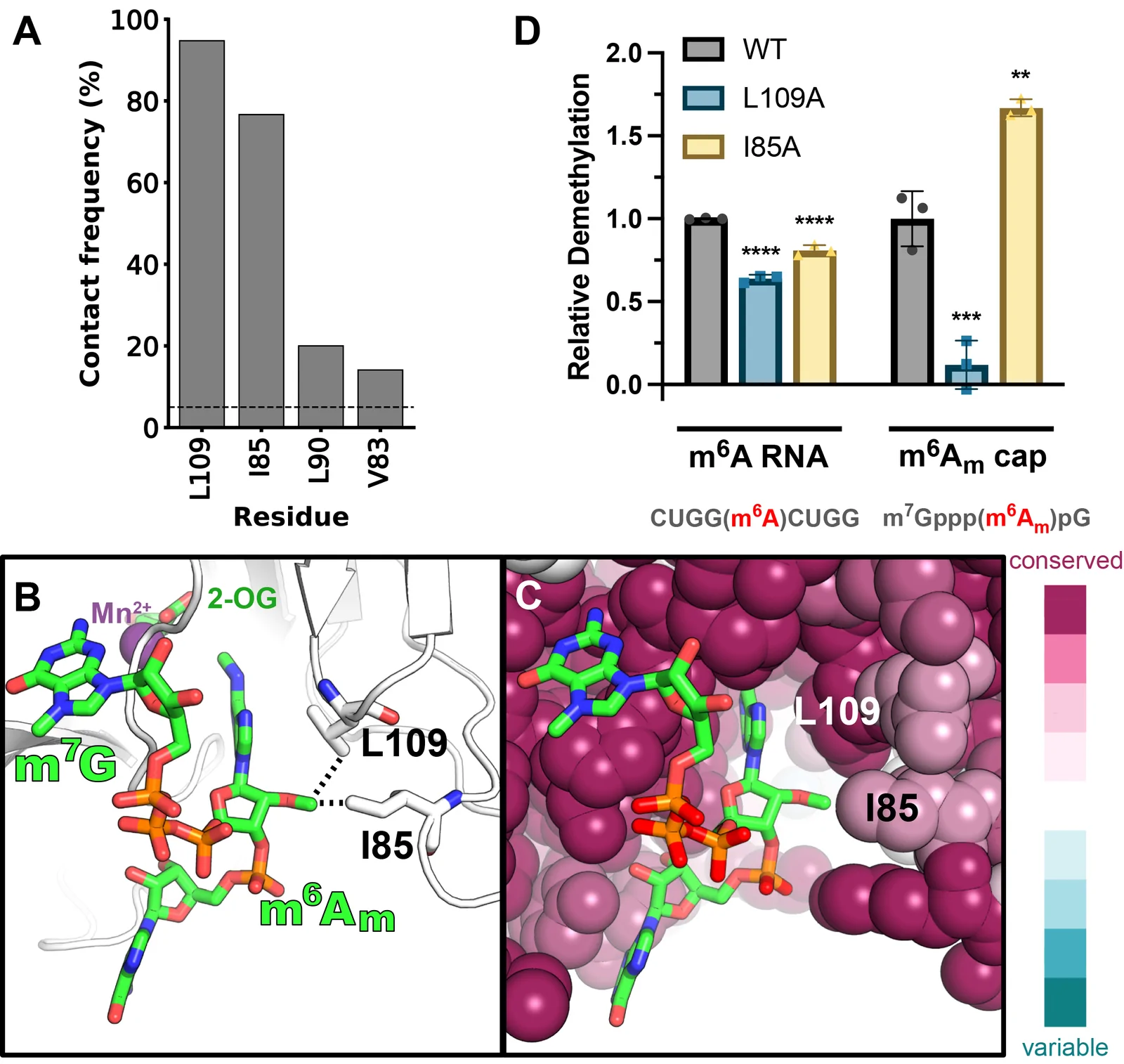
**Nonpolar FTO residues help recognize the A_m_ 2′-O-methyl group to promote m^6^A_m_ demethylation.***A,* graph showing high-frequency A_m_ 2′-O-methyl-FTO residue contacts ≤ 3.5 Å from MD simulations on the microsecond timescale. Only FTO residues with favorable contributions to computationally predicted binding affinity (ΔG ≤ −0.25 kcal/mol, Fig. S1) are considered. *B,* representative frame from MD simulations showing close interactions (*dashed lines*) of the m^6^A_m_ 2′-O-methyl group with the nonpolar FTO residues L109 and I85. *C,* representative frame from MD simulations colored by conservation showing L109 and I85 form a strongly conserved nonpolar surface located just outside the FTO active site pocket. *D,* FTO L109A and I85A mutations show altered demethylation activity for m^6^A_m_ cap compared to internal m^6^A-RNA substrates. Endpoint demethylation activity assays were conducted in triplicate with 2 μM CUGG(m^6^A)CUGG or m^7^Gppp(m^6^A_m_)pG and 1 μM FTO enzyme. Statistical significance was calculated using a one-way ANOVA with Dunnett’s multiple comparisons test (∗∗*p* ≤ 0.01, ∗∗∗*p* ≤ 0.001, ∗∗∗∗*p* ≤ 0.0001); individual data points are shown for each independent assay replicate (n = 3) and bar graphs are shown as mean values ± SD. FTO, fat mass and obesity-associated protein; m^6^A_m_, *N*^6^,2′-O-dimethyladenosine; MD, molecular dynamics; A_m_, 2′-O-methyladenosine.

## Discussion

Previous biochemical studies have shown that FTO has a clear intrinsic preference for “erasing” m^6^A_m_ modifications located at the 5′ end of mRNA, as compared to internal m^6^A modifications (26). While the 6mA-ssDNA–bound FTO structure determined in 2019 provides the structural basis for m^6^A base recognition in the active site (37), it remains entirely unknown how FTO recognizes the eukaryotic 5′ cap structure and m^6^A_m_ 2′-O-methyl group to promote efficient 5′ m^6^A_m_ demethylation on mRNA. In this work, using MD simulations we identified conserved, aromatic FTO residues H232 and W278 as key surfaces for high-frequency interaction with the m^7^G base of the m^6^A_m_ cap structure. Likewise, MD simulations predict that nonpolar FTO residues L109 and I85 form a key interaction surface for the m^6^A_m_ 2′-O-methyl group. *In vitro* activity assays validate the structural predictions from MD, and show that mutating FTO residues H232, W278, or L109 to alanine selectively impairs 5′ m^6^A_m_ demethylation, with only minimal effects on internal m^6^A demethylation. Together, our MD and biochemical data provide the first atomic-level insights into how FTO recognizes the m^7^G base (H232/W278) and the 2′-O-methyl group (L109/I85) of 5′ m^6^A_m_-modified mRNAs to promote selective m^6^A_m_ demethylation.

It is worth noting that these FTO mutations are unlikely to substantially affect protein folding, stability, or general catalytic activity. With the exception of Y220A, all of the other tested FTO mutations can efficiently demethylate internal m^6^A modifications (Figs. 2*A* and 4*D*), and H232 and W278 mutants maintain near-WT levels of activity on m^6^A_m_ substrates lacking m^7^G (Fig. 3*A*); these observations directly show that the mutations do not significantly impair overall enzyme folding, metal co-ordination, or the general catalytic functions of the enzyme. Consistent with this, mutated positions including H232, W278, and N302 are highly variable across the AlkB homolog family of enzymes (Fig. S8), suggesting substitutions there are not likely to strongly impact enzyme fold or function. Furthermore, the monomeric size exclusion peaks for recombinant WT and nearly all mutant FTO constructs are very similar, suggesting no large changes in overall protein size and/or fold due to the mutations (Fig. S9). Although FTO residue Y220 was also identified as a high-frequency site of cap interaction, the majority of MD conformations with Y220–m^7^G interactions show either Y220 hydrogen bonding with the m^7^G ribose, or the m^6^A_m_ base in a catalytically incompetent position when the m^7^G base stacks with Y220. Biochemical experiments show that Y220 mutation impairs both m^6^A and m^6^A_m_ demethylation, suggesting Y220 may help promote interactions with RNA that are not cap-specific, and/or may be important for FTO stability/folding more generally, which would be consistent with the somewhat shifted size exclusion chromatography profile for recombinant FTO Y220A (Fig. S9).

The MD simulations show m^7^G and the 5′ cap dynamically sampling multiple surfaces of FTO, with conserved residues H232 and W278 recognizing the m^7^G group through multiple predicted interaction modes. Analysis of the MD-predicted structures suggests that both of these residues can interact simultaneously with the cap, with W278 engaging the m^7^G base while H232 engages the m^7^G ribose (Fig. S4*A*), or that either residue can independently engage the m^7^G base (Fig. 1, *D* and *E*), providing a potentially redundant mechanism that promotes cap recognition across multiple conformational states. Additionally, W278 is located on a flexible loop of FTO, and MD suggests that W278 can in some instances come close enough to H232 for both of these residues to stack simultaneously with the m^7^G base of the cap (Fig. S10*A*). Therefore, another possibility consistent with our experimental data is that FTO H232 and W278 could sandwich the m^7^G base, making dual cation-π interactions that specifically recognize the 5′ cap structure. This mode of m^7^G recognition involving both FTO H232 and W278 would be similar to how canonical cap-binding proteins, such as the cap-binding complex (CBC; CBP20/80) (61) or eIF4E (62), recognize the mRNA cap—using a sandwich of aromatic residues (*e.g.,* Tyr/Tyr in the case of CBC and Trp/Trp in the case of eIF4E) to interact with the positively charged m^7^G group (Fig. S10*B*). Although our combined MD and biochemical experiments clearly demonstrate the importance of FTO H232 and W278 in m^7^G recognition to promote m^6^A_m_ demethylation, the MD simulations do not identify a single, well-defined binding conformation that is predominantly responsible for cap recognition by FTO, but instead suggest multiple plausible interaction modes involving H232 and W278.

Interestingly, computationally observed m^7^G recognition modes where m^7^G stacks with H232 (as in Fig. 1*D*) may explain a puzzling piece of biochemical data from the literature. Recently it was shown that *N*2,*N*2,7-trimethylguanosine (m^2,2,7^G)-capped RNAs, like those utilized during splicing, are entirely resistant to m^6^A_m_ demethylation by FTO (27); this is surprising because removal of m^7^G entirely only reduces m^6^A_m_ demethylation activity by 50% (26). But when m^7^G stacks with H232 as shown in Figure 1*D*, the N2 nitrogen of guanosine points directly toward the key metal binding active site residue D233. Dimethylation of the N2 atom, as in m^2,2,7^G, would sterically clash with D233 and be predicted to disrupt metal binding and inactivate FTO, consistent with the experimental observation that m^2,2,7^G-capped RNAs have no detectable m^6^A_m_ demethylation activity with FTO.

Our combined MD and biochemical studies reveal key, conserved residues on the surface of FTO that recognize the eukaryotic 5′ cap structure to promote efficient m^6^A_m_ demethylation on mRNA. These results provide a structural basis for FTO’s ability to distinguish 5′ m^6^A_m_ modifications from internal m^6^A RNA modifications, and suggest that dynamics of FTO residues and/or the 5′ cap may be important for selective cap recognition. Finally, we propose that engineered FTO mutants that selectively impair m^6^A_m_ demethylation (*e.g.*, H232A/W278A or H232A/W278A/L109A), which are further validated as separation-of-function tools by recent work from our lab (63), could be used in cell-based studies to dissect the distinct biological roles of FTO-mediated m^6^A_m_ *versus* m^6^A demethylation. Although many cellular functions of FTO have been attributed to m^6^A demethylation, increasing evidence supports cap-proximal m^6^A_m_ as an important physiological FTO substrate. For example, regulation of snRNA m^6^A_m_ has been linked to snRNA biogenesis and alternative splicing (27), and more recently to opioid reward and tolerance *in vivo* (64); quantitative studies in acute myeloid leukemia have also questioned the extent of FTO-mediated mRNA m^6^A demethylation and the importance of FTO for acute myeloid leukemia cell survival (60). Therefore, these separation-of-function variants could help define the biologically relevant RNA targets of FTO and clarify how its substrate selectivity contributes to cellular and physiological functions.

## Experimental procedures

### Model construction

An all-atom model of FTO in complex with the small 5′ cap molecule m^7^Gppp(m^6^A_m_)pG was developed based on PDB: 5ZMD (37). Missing loop segments in FTO were modeled using AlphaFold2 (65). The m^7^Gppp(m^6^A_m_)pG cap structure was built with xleap (66) using residue templates from OL3 (67). The m^6^A_m_ base was aligned into the FTO catalytic pocket based on the 6mA orientation in the FTO-6mA-ssDNA structure. Crystallographic water molecules were maintained. PDB: 2PQR (68) was used to add hydrogen atoms to FTO appropriate for pH 7.0. Local ions were placed around the complex with cionize (69), and the complex was immersed in a 125 Å^3^ box of optimal point charge (70) water containing 150 mM NaCl.

### Model parameterization

FTO was treated with the ff19SB (71) force field. Manganese(II) coordination was parameterized using MCPB.py (72) from AmberTools25 (66, 73). The 2-oxoglutarate cofactor was treated with gaff2 (74) parameters and AM1-BCC charges assigned by antechamber (66). A custom residue was constructed for m^7^Gppp(m^6^A_m_)pG. Parameters were taken from OL3 (67) based on analogy to a GAG sequence, applying updated phosphate oxygen radii (75). Methyl and triphosphate parameters were taken from gaff2 (74). Missing parameters for the methyl-purine moiety in m^7^G (two angles and an improper dihedral), as well as residue charges, were taken from the modified nucleoside force field (76), which covers methylated RNA.

### MD simulations

MD simulations in the isothermal-isobaric (NPT) ensemble were performed with pmemd.cuda (SPFP precision model) in AMBER24 (77). The system was brought to a local energy minimum using 2500 steepest descent cycles, then 2500 conjugate gradient cycles. The temperature was raised from 60 K to 310 K over a period of 5 ns, then production sampling was carried out for 1 μs, saving frames every 1 ps. Dynamics were propagated with a timestep of 2.0 fs, enabled by constraining bonds to hydrogen with the SHAKE algorithm. Nonbonded interactions were partitioned into long-and short-range components based on a cutoff of 8.0 Å. System temperature was maintained at a target of 310 K with the Langevin thermostat using a collision frequency of 1.0 ps^−1^, reinitializing the random number seed upon each simulation restart to avoid synchronization artifacts (78). System pressure was maintained at a target of 1.0 bar *via* isotropic scaling with the Berendsen barostat using a relaxation time of 1.0 ps.

### Trajectory analysis

MD simulation trajectories were analyzed using VMD 1.9.4 (69). Pairwise contacts between FTO and m^7^Gppp(m^6^A_m_)pG were defined as being atoms ≤ 3.5 Å to filter for the most relevant interactions. Only residues contributing meaningfully to FTO-m^7^Gppp(m^6^A_m_)pG binding affinity were considered for contact analysis. Binding affinity was estimated using MM/GBSA (79, 80) calculations, performed using mmpbsa.py (81) in AmberTools25 (66, 73). MM/GBSA used the Onufriev *et al.* (82) GB model II (igb = 5) with the single-trajectory protocol, in which the complex, receptor, and ligand energies are evaluated from the same trajectory. Ionic strength was set to 150 mM, and ΔG values were decomposed on a per-residue basis for each trajectory. Treatment of manganese(II) in mmpbsa.py required modification of the mdread2.F90 source file to assign the GB radius of the element.

### FTO WT and mutant expression vector construction

A codon optimized gblock of a human FTO (32–505) construct was obtained from Integrated DNA Technologies and cloned into a pET28a bacterial expression vector with N-terminal 6xHis tag. The FTO single point mutations were introduced into the 6xHis-FTO (32–505) construct using whole plasmid PCR site-directed mutagenesis.

### Protein expression and purification

FTO WT and mutant expression vectors were transformed into *Escherichia coli* BL21(DE3) cells. Twenty milliliters of an overnight culture was added to 2 L of LB media for growth at 37 °C with shaking at 200 rpm. When the absorbance at 600 nm (A_600_) reached approximately 0.6, cells were induced with 1 mM IPTG for 18 h at 18 °C with shaking at 200 rpm. Centrifugation at 4100*g* was used to harvest cells and pellets were flash-frozen and stored at −70 °C. Pellets were thawed and resuspended in lysis buffer (25 mM Tris–Base pH 8.0, 300 mM NaCl, 5 mM 2-mercaptoethanol), lysed by sonication, and centrifuged at 24000*g* for 45 min. Soluble His-tagged FTO was purified using HisPur Ni-NTA affinity resin (Thermo Fisher Scientific) at 4 °C and eluted in 25 mM Tris–HCl pH 7.5, 300 mM NaCl, 400 mM imidazole. Protein was buffer exchanged into 25 mM Tris–HCl pH 7.5, 25 mM NaCl and then purified using ion exchange chromatography with a 5 mL HiTrapQ column (Cytiva) and linear gradient into 25 mM Tris–HCl pH 7.5, 1 M NaCl. Further purification was done with size-exclusion chromatography using a Superdex 200 pg 16/60 gel filtration column (Cytiva) in 10 mM HEPES pH 7.0, 50 mM KCl. Protein fractions were visualized using SDS-PAGE and purified FTO fractions were combined, concentrated, flash-frozen, and stored in size-exclusion buffer with 10% glycerol at −70 °C.

### FTO demethylation assays

*In vitro* FTO demethylation activity assays were performed by incubating 2 μM substrate (m^7^Gppp(m^6^A_m_)pG, m^7^Gppp(m^6^A_m_)pCpUpGpG, or CUGG(m^6^A)CUGG) with either 1 μM FTO or 0.1 μM FTO in demethylation reaction buffer (50 mM HEPES pH 7.0, 150 mM KCl, 75 μM (NH_4_)_2_Fe(SO_4_)_2_·6H_2_O, 300 μM 2-oxoglutarate, and 2 mM ascorbic acid). Reactions were initiated by addition of a 2X substrate mixture in reaction buffer to a 2X enzyme and cofactor mixture in reaction buffer and mixed with pipetting. Reaction time points were quenched by addition of EDTA to a final concentration of 1 mM.

### RNA nucleoside analysis and quantification

Quenched reaction samples with substrate m^7^Gppp(m^6^A_m_)pCpUpGpG were first decapped using NEB mRNA decapping enzyme (M0608S) for 1 h. All quenched reaction samples were digested with NEB Nucleoside Digestion Mix (M0649S) for 1 h (CUGG(m^6^A)CUGG) or 18 h (m^7^Gppp(m^6^A_m_)pG or m^7^Gppp(m^6^A_m_)pCpUpGpG). Following RNA digestion into single nucleosides, for reaction samples with 1 μM FTO, protein was precipitated by addition of 20% trichloroacetic acid. Samples were then centrifuged and supernatant was collected for LC-MS analysis. Analysis of demethylation status was performed on an Agilent Bio-Inert 1260 Infinity II UHPLC system with Infinity Lab LC/MSD iQ. Product nucleoside concentrations were quantified by comparison of LC-MS peak areas with linear calibration curves generated using synthetic nucleoside standards of known concentration. Separation of individual RNA nucleosides was conducted using an Agilent Zorbax SB-Aq Rapid Resolution HD (2.1 × 100 mm, 1.8 μm particle size) using mobile phase-containing 0.1% formic acid (A) and 100% acetonitrile, 0.1% formic acid (B) and then detected by mass spectrometry in positive ionization mode. The gradient was as follows for detection of m^6^A at 0.480 ml/min (m/z = 282, 3.5 min) and A (m/z = 268, 1.6 min): 0 to 1.0 min, 100% A; 1.0 to 4.25 min, to 85% A/15% B; 4.25 to 5.0 min, to 25% A/75% B; 5.0 to 7.0 min, 25% A/75% B; 7.0 to 7.1 min, to 100% B; 7.1 to 11.75 min, 100% B. The gradient was as follows for detection of m^6^A_m_ at 0.450 ml/min (m/z = 296, 3.65 min) and A_m_ (m/z = 282, 3.5 min): 0 to 1.0 min, 100% A; 1.0 to 1.1 min, to 40% A/60% B; 1.1 to 5.0 min, to 28% A/72% B; 5.0 to 5.1 min, to 100% B; and 5.1 to 8.0 min, 100% B.

### Michaelis–Menten kinetic analysis

Michaelis–Menten kinetic analyses for WT FTO and H232A/W278A FTO were performed similarly as described above for FTO demethylation assays, but using 0.2 μM FTO and m^7^Gppp(m^6^A_m_)pCpUpGpG substrate, with substrate concentration varied across the kinetic series (1–16 μM substrate with WT FTO; 2–25 μM substrate with H232A/W278A FTO). Following quenching, decapping, nucleoside digestion, and UHPLC-MS quantification as described above, initial velocities were determined from linear fits of product concentration *versus* reaction time. Initial velocities were plotted as a function of substrate concentration and fit by nonlinear least-squares regression to the *k*_cat_ Michaelis–Menten model in GraphPad Prism (https://www.graphpad.com/; E_t_ constrained to 0.2 μM) to determine *k*_cat_ and *K*_M_. Three independent kinetic experiments were performed for each enzyme, with each independent experiment comprising a complete substrate concentration series. For graphical presentation and determination of kinetic parameters, mean initial velocities at each substrate concentration were fit to the Michaelis–Menten model (Fig. S6); for statistical comparison of WT *versus* H232A/W278A kinetic parameters, each independent kinetic experiment was also fit separately to obtain individual *k*_cat_ and *K*_M_ estimates, which were then compared using two-tailed paired *t*-tests (Table S1).

### RNA substrate preparation

9-mer m^6^A RNA, CUGG(m^6^A)CUGG, was synthesized by Integrated DNA Technologies. Cap m^6^A_m_ RNA substrates (m^7^Gppp(m^6^A_m_)pG and m^7^Gppp(m^6^A_m_)pCpUpGpG) were synthesized as previously described for similar RNA cap molecules (83).

## Data availability

All data supporting the conclusions of this article are contained within the article and supporting information. Additional data are available from the corresponding authors upon reasonable request (jhadden@udel.edu; mugridge@udel.edu).

## Supporting information

This article contains supporting information.

## Conflict of interest

The authors declare that they have no conflicts of interest with the contents of this article.
