# Supplementary material for: RNA Demethylase FTO Uses Conserved Aromatic Residues to Recognize the mRNA 5′ Cap and Promote Efficient m6Am Demethylation
Source: J Biol Chem. 2026 Sep 17;302(11):113573. doi: 10.1016/j.jbc.2026.113573 (PMC13693085; doi:10.1016/j.jbc.2026.113573)
Supplement: Supplementary file 1 [file mmc1.pdf]

SUPPORTING INFORMATION

RNA Demethylase FTO Uses Conserved Aromatic Residues to Recognize the mRNA 5′ Cap and Promote Efficient m^6A_m Demethylation

Brittany Shimanski[1†], Juan F. Marin[1†], Marcin Warminski[2], Kasun Dilshan Abeyrathne Eluwawalage[1], Lucas O. Calzini[1], Riley M. McKeon[1], Joanna Kowalska[2], Jacek Jemielity[3], Jodi A. Hadden-Perilla[1,*], Jeffrey S. Mugridge[1,*]

[1] *Department of Chemistry & Biochemistry, University of Delaware, Newark, DE 19716, United States*

[2] *Division of Biophysics, Institute of Experimental Physics, Faculty of Physics, University of Warsaw, 02-089 Warsaw, Poland*

[3] *Centre of New Technologies, University of Warsaw, 02-089 Warsaw, Poland*

[†] These authors contributed equally to this work

Corresponding authors:

***Jodi A. Hadden-Perilla**. Department of Chemistry & Biochemistry, University of Delaware, Newark, DE 19716, United States; orcid.org/0000-0003-4685-8291; Email: jhadden@udel.edu

***Jeffrey S. Mugridge**. Department of Chemistry & Biochemistry, University of Delaware, Newark, DE 19716, United States; orcid.org/0000-0002-1553-3008; Email: mugridge@udel.edu

Supporting Information

This Supporting Information file contains Figures S1–S10 and Table S1.

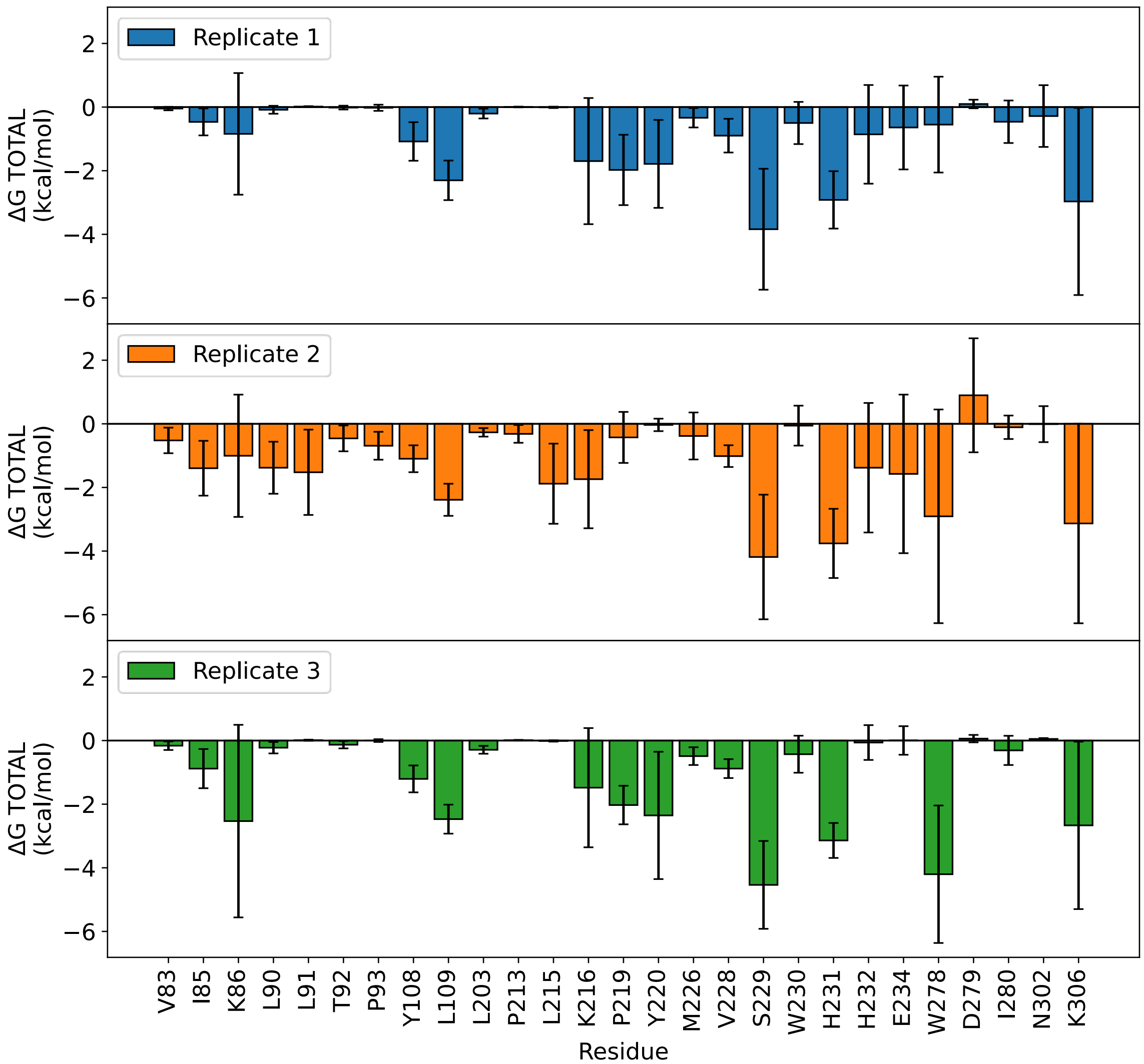

Figure S1. FTO-m^{7}Gppp(m^{6}A$_m$)pG binding energies determined from MD simulations. Average per-residue contributions to the total Gibbs free energy of binding **(**ΔG, kcal/mol) calculated using the molecular mechanics/generalized Born surface area (MM/GBSA) method. Error bars represent standard deviation. FTO residues are included here if the magnitude of their contribution was > 0.25 kcal/mol in at least one replicate. Negative values indicate favorable contributions to binding. Values are averaged over one million MD frames per replicate.

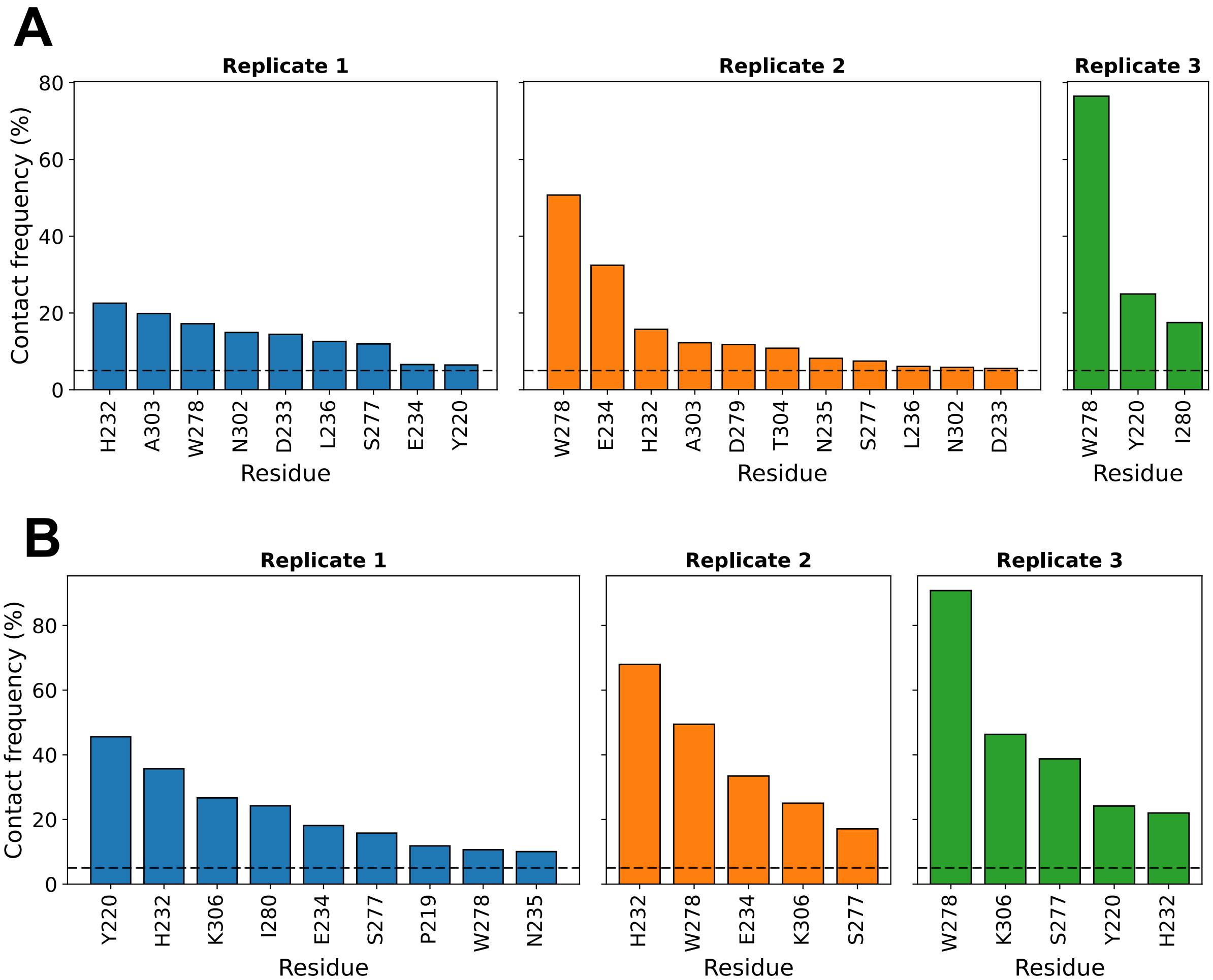

Figure S2. FTO-m^{7}G contact frequencies determined from MD simulations. Contact between FTO residues and m^{7}G was defined as atomic distances $\leq$ 3.5 Å. FTO residues are included here if their m^{7}G contact frequency exceeded 5% of MD trajectory frames; percentages were calculated out of one million frames per replicate. Panel A lists FTO residues contacting the m^{7}G base, and panel B lists FTO residues contacting the m^{7}G ribose for each MD replicate.

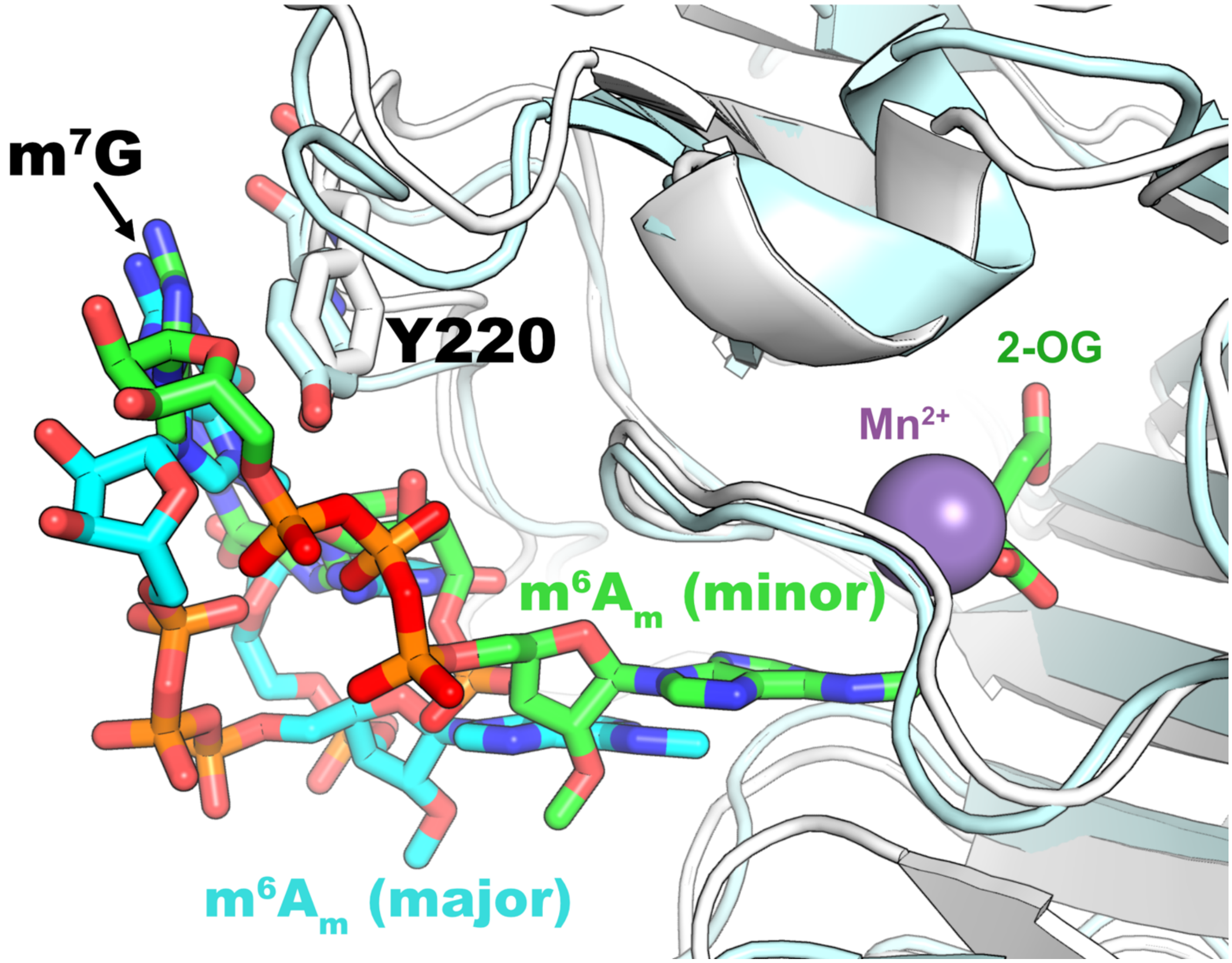

Figure S3. MD simulations suggest that Y220-m^{7}G base stacking is not conducive to m^{6}A$_m$ demethylation. During a preliminary run of Replicate 2, m^{7}G lost contact with FTO residues, leading to destabilization of the m^{7}Gppp(m^{6}A$_m$)pG binding mode and shifting of the m^{6}A$_m$ base away from the active site metal. The MD simulation was restarted from a frame in which appropriate m^{7}G and m^{6}A$_m$ interactions remained in place to continue sampling the catalytically relevant complex. However, the preliminary run demonstrated that Y220-m^{7}G base stacking was favored when the m^{6}A$_m$ base was only shallowly positioned in the active site (cyan 'major' conformation above, in which the m^{6}A methyl group is ~8 Å from active site metal; corresponds to light cyan FTO cartoon). A limited number of trajectory frames preceding the ligand shift showed m^{7}G-Y220 interaction modes with the m^{6}A$_m$ base positioned deeper within the active site, in a conformation more consistent with demethylation catalysis (green 'minor' conformation above, in which the m^{6}A methyl group is ~4 Å from active site metal; corresponding to white FTO cartoon). These results suggest that Y220-m^{7}G base stacking may promote m^{6}A$_m$ cap binding modes that are ineffective for catalysis.

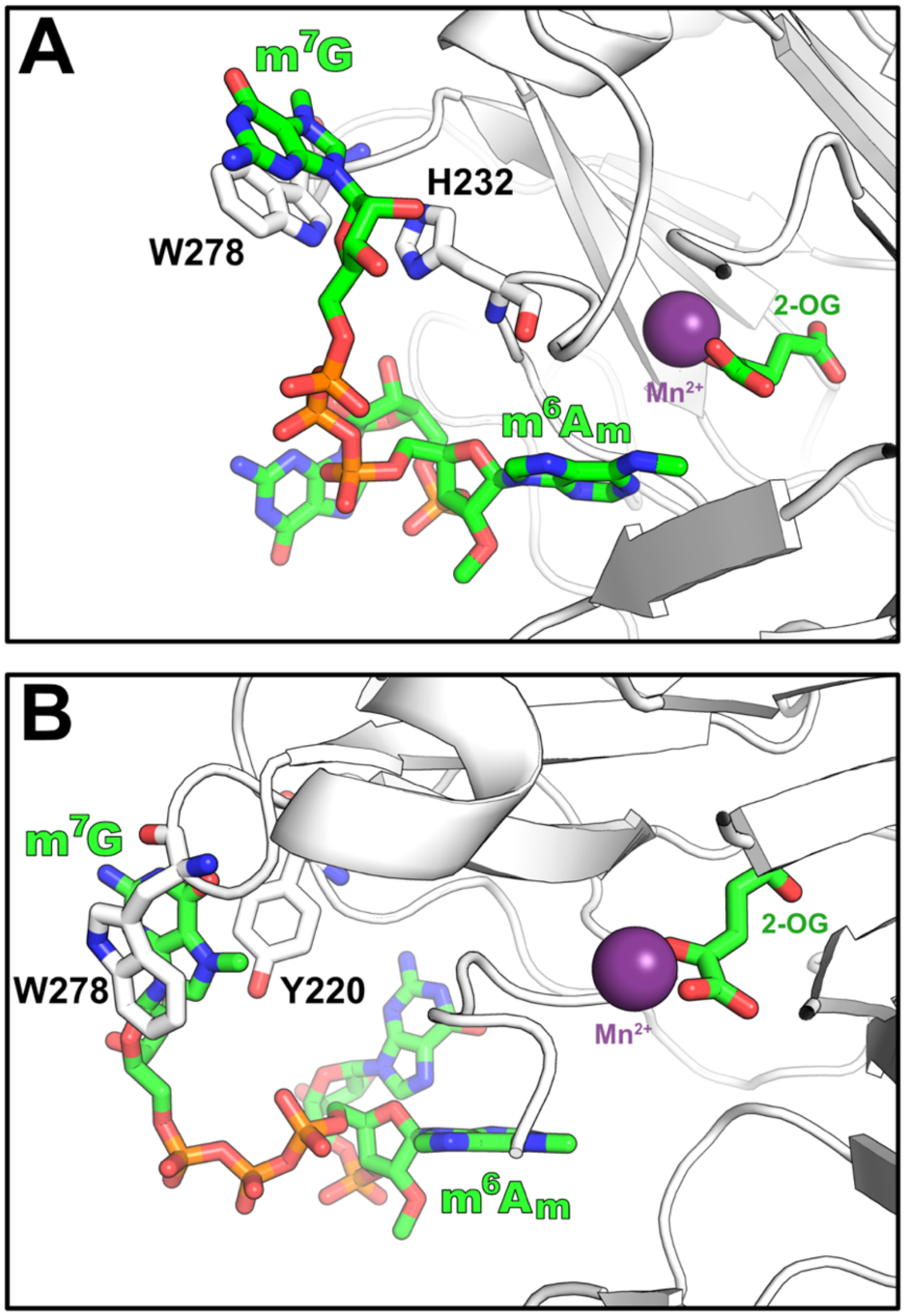

Figure S4. FTO-m^{7}G binding modes with dual interactions observed in MD simulations. **(A)** Dual interaction mode where H232 engages the m^{7}G ribose through CH-π/van der Waals interactions, and W278 stacks or forms cation-π interactions with the m^{7}G guanosine base. **(B)** Dual interaction mode observed where Y220 and W278 engage the m^{7}G base in simultaneous stacking / cation-π interactions, which correlates with the m^{6}A$_m$ base being withdrawn somewhat from the active site pocket. In this particular MD frame, the m^{6}A methyl group is ~10 Å from the active site metal. The latter mode was observed during a preliminary run of Replicate 2, in which m^{7}G lost contact with FTO residues, leading to destabilization of the m^{7}Gppp(m^{6}A$_m$)pG binding mode and shifting of the m^{6}A$_m$ base away from the active site metal. The MD simulation was restarted from a frame in which appropriate m^{7}G and m^{6}A$_m$ interactions remained in place to continue sampling the catalytically relevant complex. However, the preliminary run demonstrated that Y220-m^{7}G base stacking was favored only with m^{6}A$_m$ cap binding modes expected to be ineffective for catalysis.

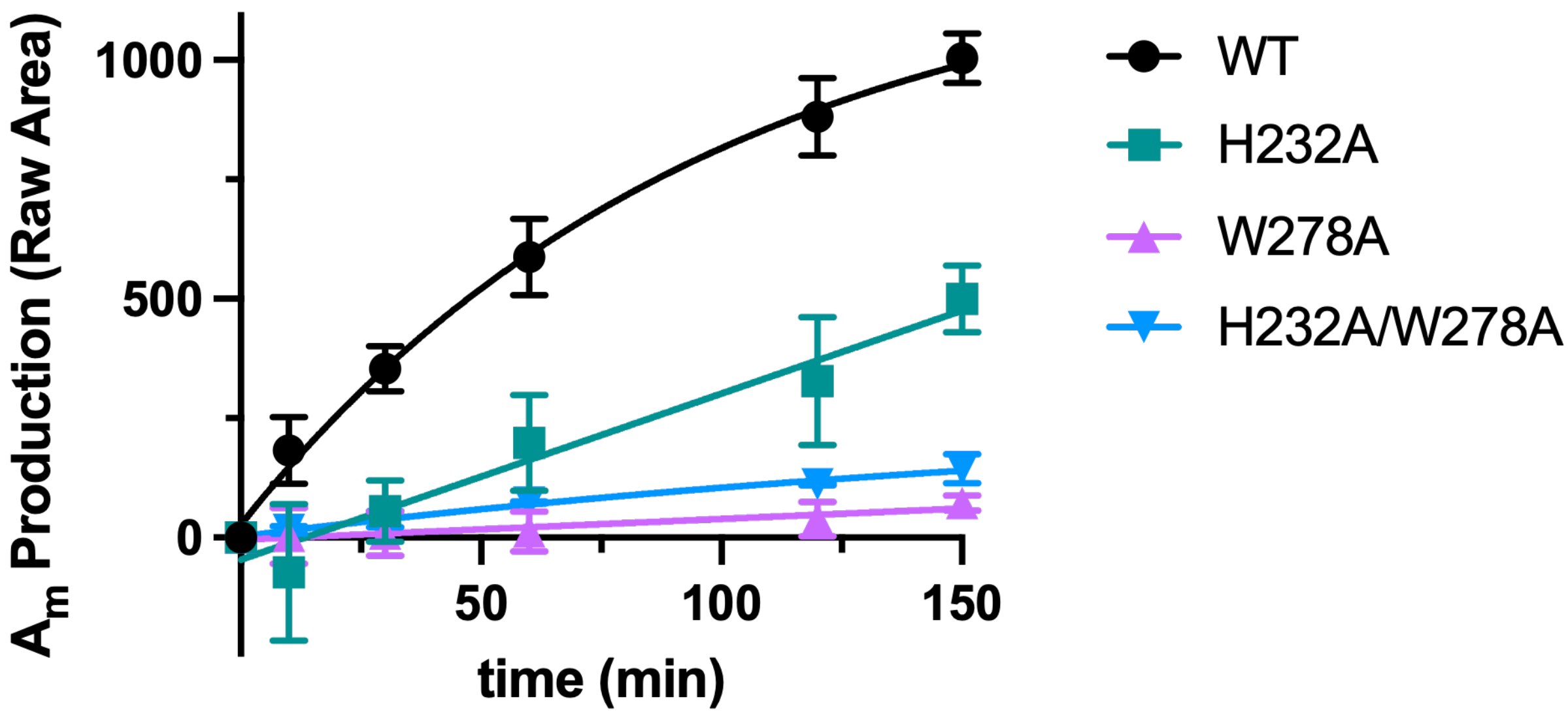

Figure S5. m^6A_m demethylation time courses with cap substrates. Mutation of conserved aromatic residues in FTO identified as m^7G interaction sites show reduced production of A_m product during demethylation reactions with model m^6A_m cap substrates. Demethylation time courses were conducted with 2 μM $m^7Gppp(m^6A_m)pG$ and 1 μM FTO enzyme in triplicate. At various time points from 0 to 150 minutes, reaction samples were quenched with EDTA, digested to single nucleosides with RNAase cocktail, and the relative amounts of A_m were quantified by UHPLC-MS. The integrated MS area (raw area) is plotted versus time and data are shown as mean values ± SEM (n = 3).

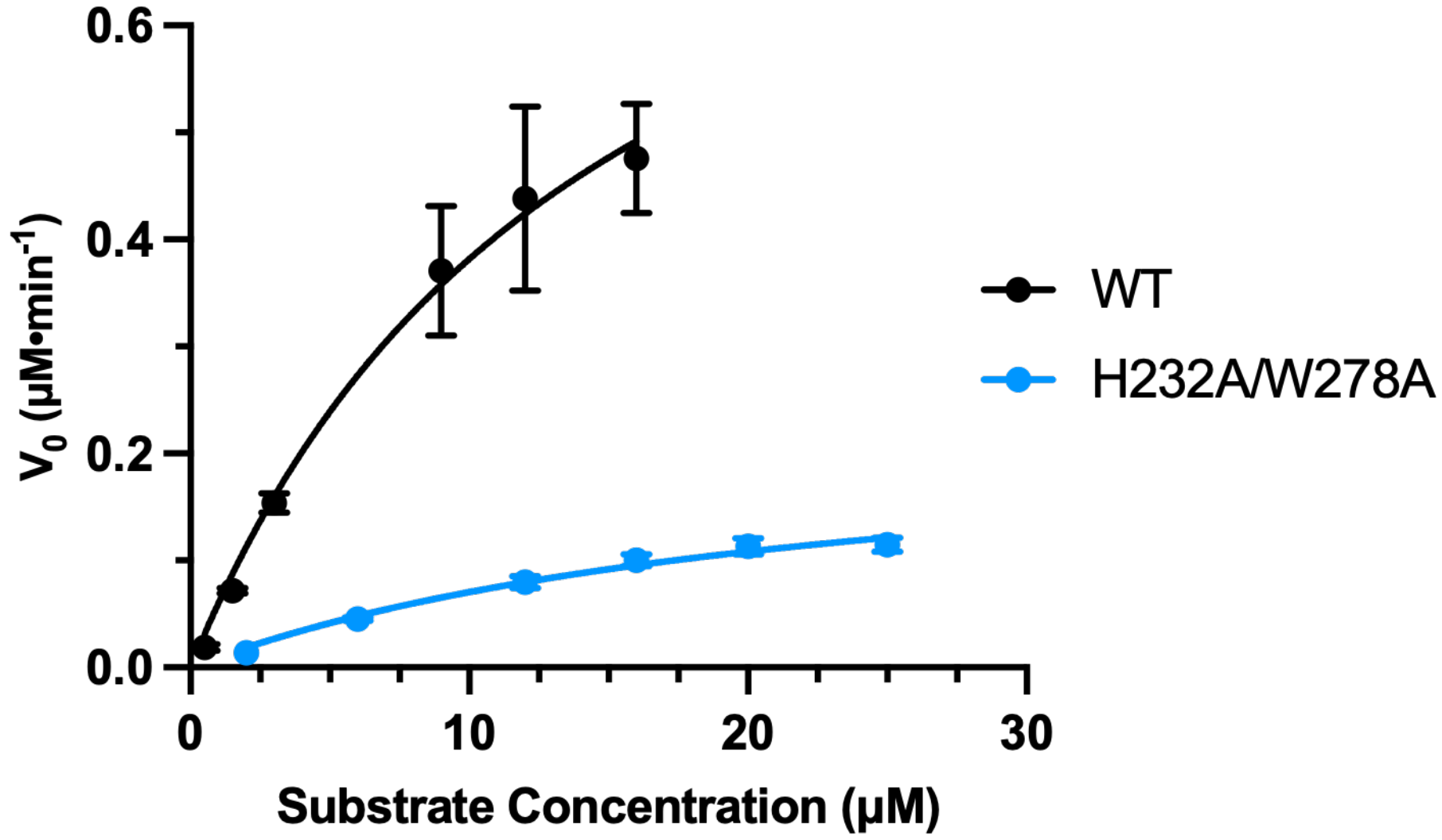

Figure S6. Michaelis-Menten analysis of m^6A_m demethylation by FTO WT and H232A/W278A mutant. Michaelis-Menten kinetic analysis of FTO-mediated demethylation of m^7Gppp(m^6A_m)pCpUpGpG cap substrate by WT FTO (black) versus the H232A/W278A double mutant (blue). Data points represent mean initial velocities ± SEM from three independent kinetic experiments at each substrate concentration. Each independent experiment consisted of a separately performed complete substrate concentration series. Curves are nonlinear least-squares fits of the mean initial velocities to the Michaelis-Menten equation. Best-fit kinetic parameters and statistical comparisons between WT and H232A/W278A are reported below in **Table S1**. WT FTO kinetic data are as previously reported in Calzini et al, *bioRxiv* **2026**, *doi*:10.1101/2025.05.06.652568.

Parameter	WT FTO	H232A/W278A FTO	Change	*P* value
k_{cat} (min^{-1})	4.7 ± 1.6	1.2 ± 0.2	~3.9-fold decrease	0.048 (*)
K_M (μM)	15 ± 9	23 ± 7	~1.5-fold increase	0.186 (ns)

Table S1. Michaelis-Menten kinetic parameters of m^6A_m demethylation by FTO WT and H232A/W278A mutant. Values for k_{cat} and K_M are best-fit parameters obtained from the nonlinear least-squares fitting of the mean initial velocities from three independent kinetic experiments to the Michaelis-Menten equation (shown above in **Figure S6**). Kinetic parameters are reported as the best-fit value ± SE of the fitted parameter. For statistical comparison, each independent kinetic experiment was additionally fit separately to obtain individual k_{cat} and K_M estimates (n = 3 independent experiments per FTO construct). WT and H232A/W278A parameter estimates were then compared using two-tailed paired t-tests. Compared to WT FTO, the H232A/W278A double mutant exhibited an approximately 4-fold decrease in k_{cat} (P = 0.048, *), whereas the approximately 1.5-fold increase in K_M was not statistically significant (P = 0.186, ns); $P < 0.05$ was considered statistically significant.

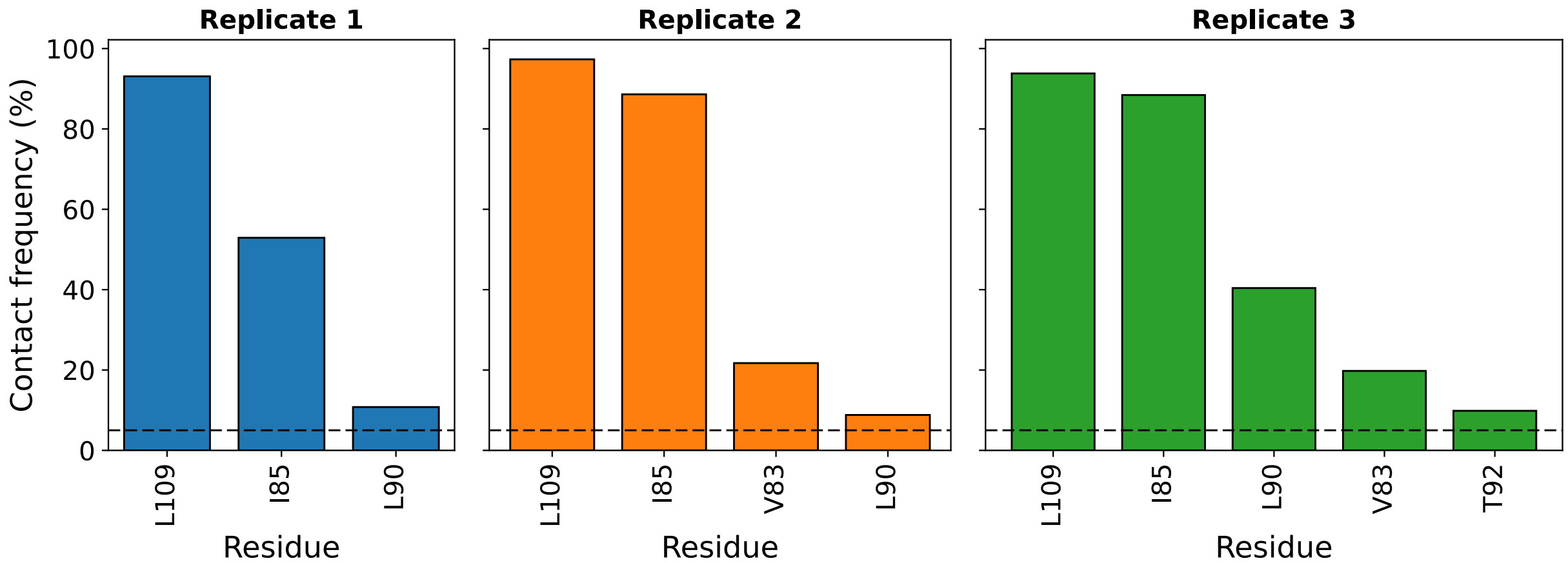

Figure S7. FTO-A_m 2′-O-methyl group contact frequencies determined from MD simulations. Contact between FTO residues and the methyl group was defined as atomic distances ≤ 3.5 Å. FTO residues are included here if their methyl group contact frequency exceeded 5% of MD trajectory frames; percentages were calculated out of one million frames per replicate.

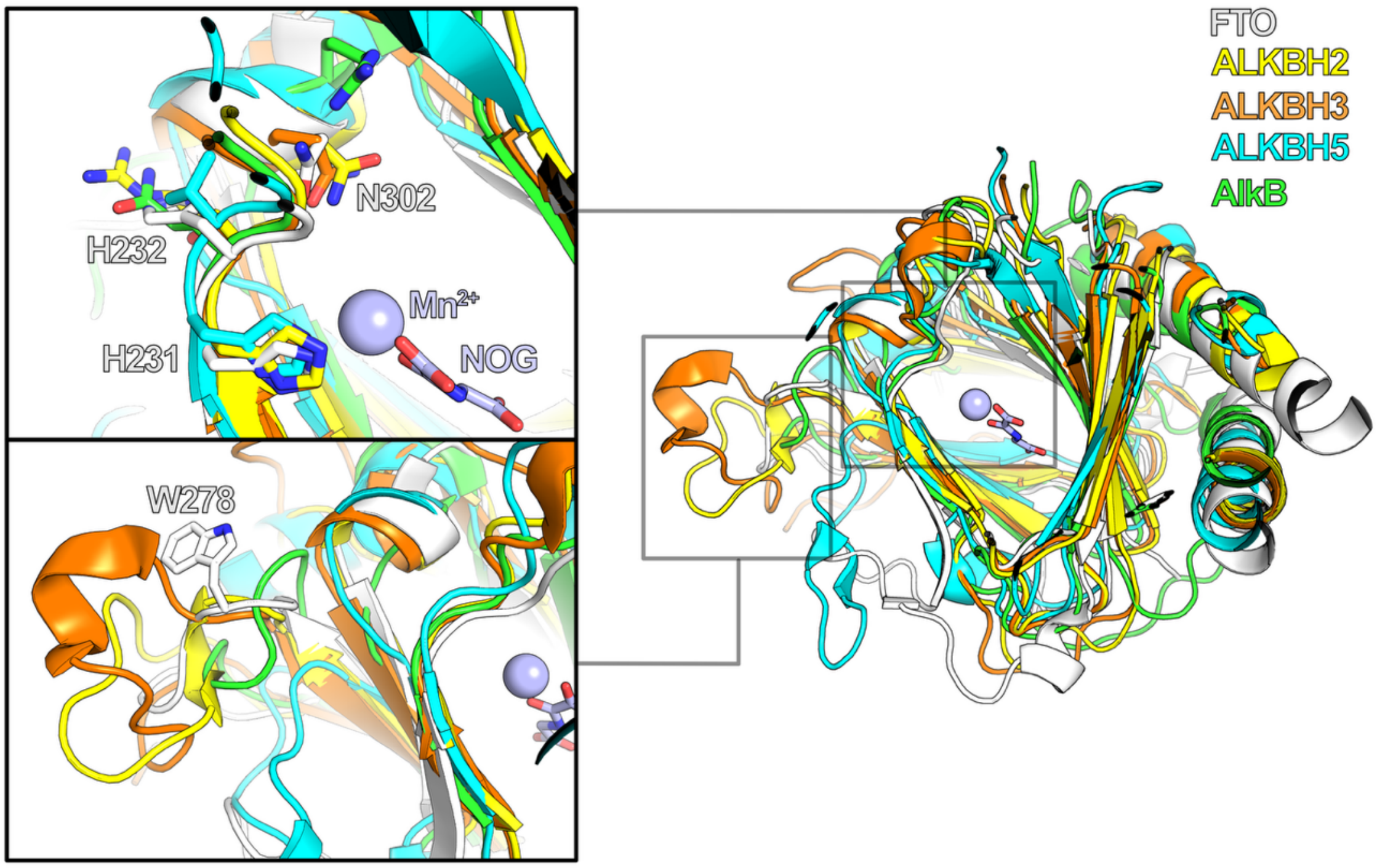

FTO	AlkBH2	AlkBH3	AlkBH5	AlkB
H232	R172	S192	V205	Q132
N302	N231	Q252	A261	R183
W278	--	--	--	--

Figure S8. Mutated FTO positions are highly variable across the AlkB homolog family. (**Top**) Structural alignments of core catalytic domains for human FTO (gray), ALKBH2 (yellow), ALKBH3 (orange), ALKBH5 (cyan), and *E. coli* AlkB (green). Insets are zoom-ins showing variable H232 and N302 positions, along with the nearby highly-conserved metal binding H231 position (upper inset); and W278 position which is a variable loop in all of the structures (lower inset). (**Bottom**) Table listing equivalent residues at the FTO H232 and N302 position across the AlkB homolog family; because W278 is located in a variable loop, structurally/sequence equivalent positions are not readily identifiable, but none of these loops contain Trp residues. The variability of these positions across the AlkB family suggests mutations at these positions are unlikely to significantly disrupt enzyme folding, stability, or general catalysis, consistent with our observations that mutations in FTO at these positions maintain robust demethylation activity on m^6A modifications.

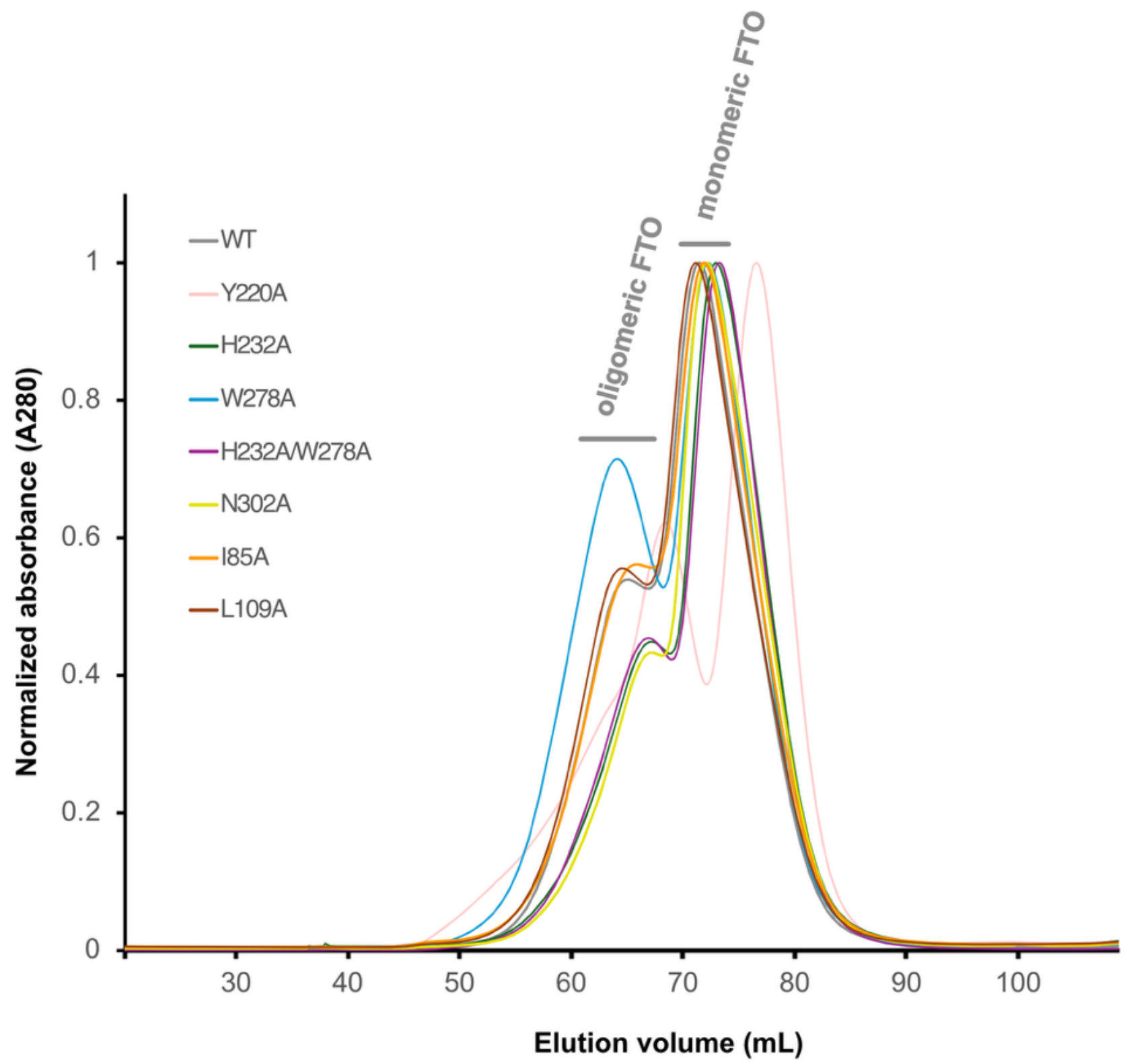

Figure S9. Size exclusion chromatograms of purified recombinant FTO WT and mutant enzymes. Overlaid, absorbance-normalized size exclusion traces for FTO variants show a very similar monomeric FTO peak for WT and nearly all FTO mutants tested. The exception is Y220A, whose monomeric SEC peak is notably shifted, possibly due to a change in the protein fold/shape/dispersity. Small differences in the elution volume for the monomeric FTO peak and differences in the oligomeric:monomeric peak ratios are likely due to prep-to-prep differences in protein loading amounts and/or differences in salt concentration carried over from the preceding ion exchange chromatography step. Only monomeric peak fractions were collected and used for biochemical experiments. The highly similar monomeric peaks observed for FTO WT and mutant variants suggest that these mutations do not induce significant unfolding, instability, or aggregation.

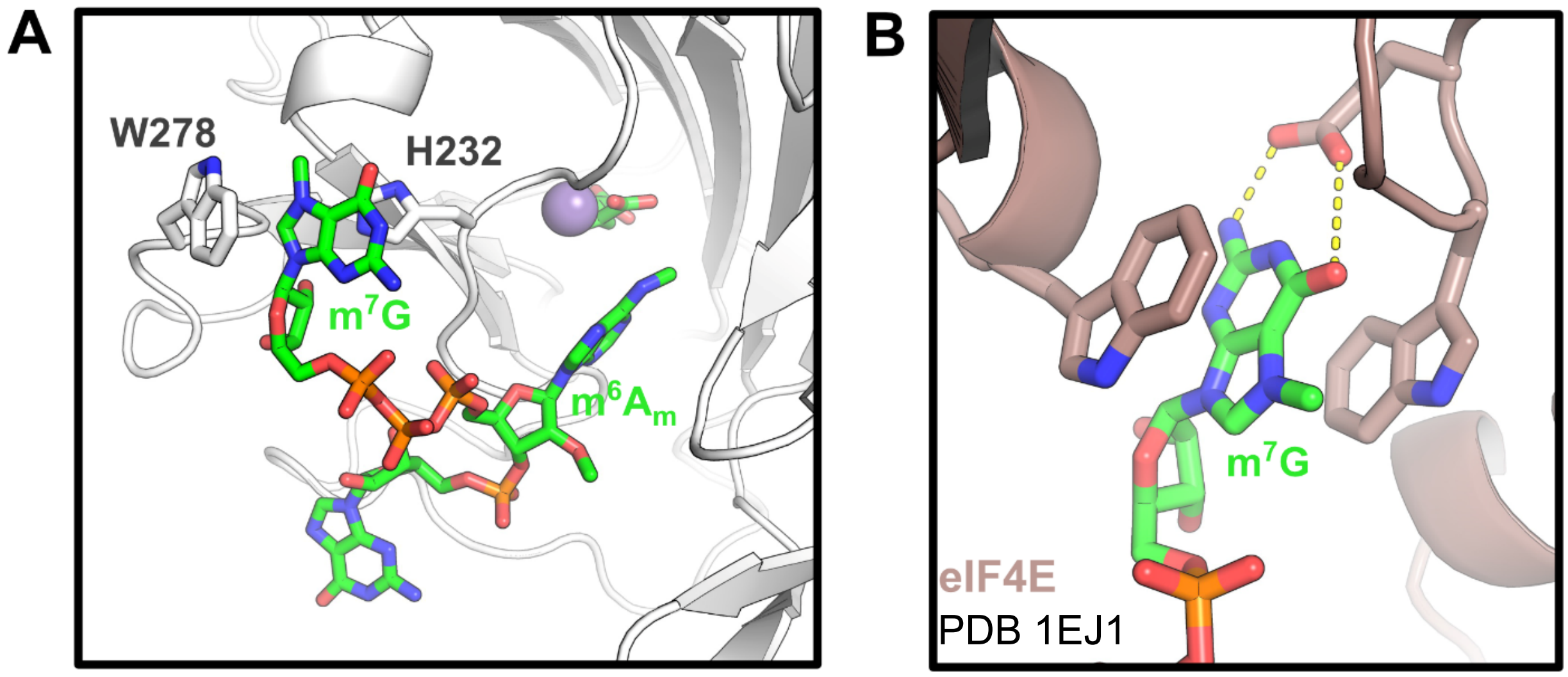

Figure S10. **Simultaneous stacking interaction of aromatic residues with m^{7}G. (A)** Representative MD frame showing m^{7}G weakly stacked between conserved aromatic FTO residues H232 and W278, which was observed only rarely during MD simulations. **(B)** Canonical cap recognition showing m^{7}G stacked between two aromatic residues of eIF4E from PDB 1EJ1.
